# The “addicted” spine

**DOI:** 10.3389/fnana.2014.00110

**Published:** 2014-10-02

**Authors:** Saturnino Spiga, Giovanna Mulas, Francesca Piras, Marco Diana

**Affiliations:** ^1^Department of Animal Biology and Ecology, University of CagliariCagliari, Italy; ^2^“G.Minardi” Laboratory of Cognitive Neuroscience, Department of Chemistry and Pharmacy, University of SassariSassari, Italy; ^3^Department of Natural Science and the Territory, University of SassariSassari, Italy

**Keywords:** spines, long thin, learning, dopamine, nucleus accumbens

## Abstract

Units of dendritic branches called dendritic spines represent more than simply decorative appendages of the neuron and actively participate in integrative functions of “spinous” nerve cells thereby contributing to the general phenomenon of synaptic plasticity. In animal models of drug addiction, spines are profoundly affected by treatments with drugs of abuse and represent important sub cellular markers which interfere deeply into the physiology of the neuron thereby providing an example of the burgeoning and rapidly increasing interest in “structural plasticity”. Medium Spiny Neurons (MSNs) of the Nucleus Accumbens (Nacc) show a reduced number of dendritic spines and a decrease in TH-positive terminals upon withdrawal from opiates, cannabinoids and alcohol. The reduction is localized “strictly” to second order dendritic branches where dopamine (DA)-containing terminals, impinging upon spines, make synaptic contacts. In addition, long-thin spines seems preferentially affected raising the possibility that cellular learning of these neurons may be selectively hampered. These findings suggest that dendritic spines are affected by drugs widely abused by humans and provide yet another example of drug-induced aberrant neural plasticity with marked reflections on the physiology of synapses, system structural organization, and neuronal circuitry remodeling.

## Introduction

Dendritic spines have been recognized, described and named, for the first time by Ramón y Cajal on the surface of Purkinje cells using the Golgi staining method (Cajal, 1888, 1891). While other investigators and even Golgi himself, disregarded spines as artifacts, Gray (1959) unambiguously showed that spines were sites of synaptic contact. It is now clear that dendritic spines are the main postsynaptic compartments of excitatory synapses in the brain with peculiar and distinctive morphological features.

Dendritic spines are heterogeneous in size and shape but, mostly mature ones, consist of a bulbous head and a thinner neck that connects the spine to the dendritic shaft (Wilson et al., 1983; Svoboda et al., 1996). This morphological configuration is particularly important for synaptic efficacy. In particular, dimensions of the spine head (Kirov and Harris, 1999; Holtmaat and Svoboda, 2009), rather than the neck, realistically reflect the observed differences in synaptic strength (Harris and Stevens, 1988). The neck constriction might serve to isolate metabolic events in the vicinity of activated synapses without significantly influencing the transfer of synaptic charge to the parent dendrite (Harris and Stevens, 1988) and thus favoring “local” changes in the number and shape of spines during synaptic plasticity (Engert and Bonhoeffer, 1999). Indeed, individual spines may represent partially autonomous compartments with a cytoskeleton composed mostly of F-actin, and may hold numerous specialized organelle such as the smooth endoplasmic reticulum, which in the largest spines forms the “spine apparatus” (Gray, 1959) with polyribosomes, near the base of the spine (Steward and Levy, 1982) offering the possibility of local protein synthesis.

At the ultrastructural level, the spine head is characterized by an electron-dense matrix of receptors and supporting proteins collectively known as the postsynaptic density (PSD; Yamauchi, 2002). This complex assembly, made of hundreds of distinct proteins (Moon et al., 1994), dynamically changes its structure and composition during development and in response to synaptic activity. The PSD contains signaling molecules including the subunits of the *N*-methyl-D-aspartate (NMDA) glutamate receptors, the a-amino-3-hydroxy-5-methyl-4-isoxazolepropionic-acid (AMPA), the subunits of Ca21/calmodulin-dependent protein kinase II (CaMKII; Kennedy et al., 1983) and synGAP, a ras GTPase-activating protein phosphorylated by CaMKII and dopamine (DA) receptors (Zhang et al., 2007). Other prominent PSD proteins are scaffold molecules, including the PSD-95 family (Cho et al., 1992), that link receptors to signaling proteins or to the cytoskeleton, thus helping organize the structure of PSDs (Kornau et al., 1995).

## Classification

Spine development is a dynamic process which includes transition from small dendritic formations to large spines and vice versa, through a series of sophisticated structural refinements (Calabrese et al., 2006). The continuous and rapid change in shape of dendritic spines is essential for short and long term plasticity (Kasai et al., 2003, 2010) and different shapes may reflect dynamically different functions (Hering and Sheng, 2001).

A pioneering classification was proposed by Peters and Kaiserman-Abramof (1970), where they distinguished three categories: stubby, thin, and mushroom spines. However, it was necessary to introduce the dendritic filopodia in this classification. In some cases, following establishment of contact with an afferent fiber, these transient structures can become a spine (Ziv and Smith, 1996; Fiala et al., 1998; Sorra and Harris, 2000). On the other hand, some author prefers to distinguish mature spines into two broad categories: large and small considering the head size (Kasai et al., 2003, 2010) emphasizing spine function.

**Figure 1:**
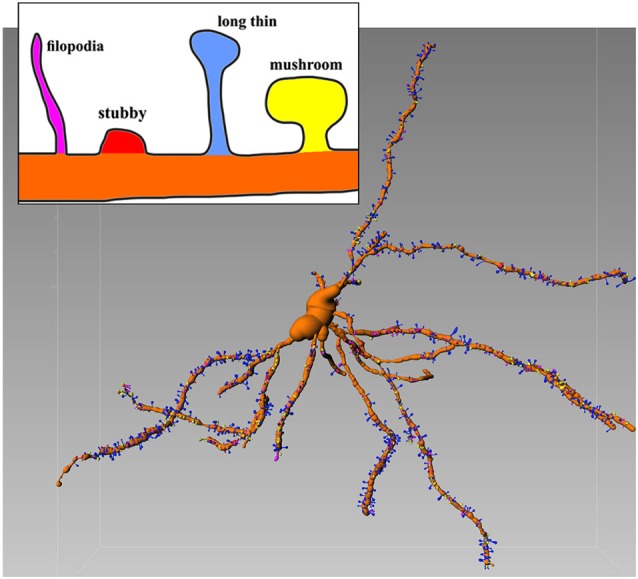
**Representative Golgi-Cox stained MSN with various spines types**. Inset shows details of different morphologies. Image is color-coded. Reconstructed with filament tracer algorithm (Imaris 7.4). Note relative abundance of blu (long thin) spines which amount for 52% of all spines (see Spiga et al., 2014 for further details).

The confocal microscope is able to detect sufficient details of the Golgi-Cox-stained neurons. In this case is possible to extract numerical information from 3D recontruction and to establish an unambiguous criterion of classification (Figure 1) which was recently introduced (Spiga et al., 2014).

## The spine of the nucleus accumbens

**Figure 2:**
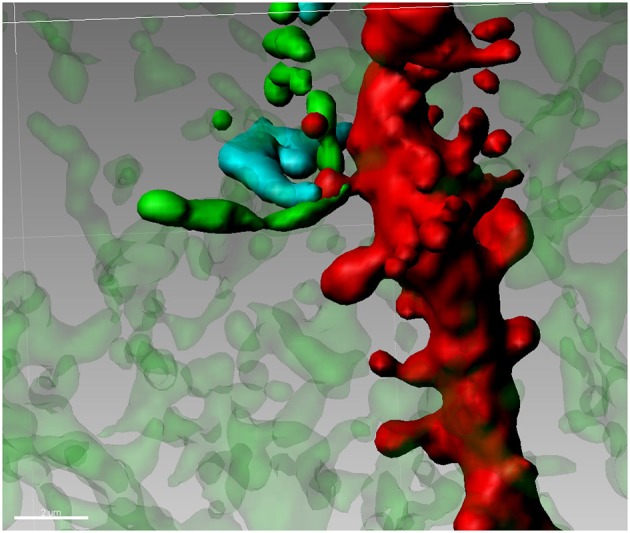
**Synaptic triad in the Nucleus Accumbens**. Tyrosine Hydroxylase-positive terminals (green) are forming a putative contact with the neck of a spine on a second order dendritic trunk (red), while the head of the same spine is reached by a Golgi-Cox impregnated fiber (blue) from an adjacent neuron.

The Medium Spiny Neuron (MSN) of the Nucleus Accumbens (Nacc) plays a central role in the integration of cortical, thalamic and mesencephalic afferents and MSNs (accounting for 90–95% of the total striatal complex) are involved in various behavioral sequelae including movement control (Björklund and Dunnett, 2007; Pissadaki and Bolam, 2013), motivation (Ostlund et al., 2014) and addiction (Diana, 2011). Terminals of DA containing neurons from the ventral tegmentum (VTA) are jumbled in a dense network of connections in many forebrain regions. Although the number of these neurons is relatively small, the projections from individual neurons are very extensive having a total axonal length (including collaterals) of roughly 74 cm with 500,000 terminals (Björklund and Dunnett, 2007; Pissadaki and Bolam, 2013) forming, in the striatum, approximately 20% of all synapses (Zhou et al., 2002, 2003). Basically, in this area every MSN is innervated by a conspicuous number of DAergic axons (Yao et al., 2008). MSNs also receive glutamate inputs from the PFC, thalamus, hippocampus (Harris and Stevens, 1989), and amygdala (Bredt and Nicoll, 2003). Accordingly, the Nacc plays a central role in the integration of cortical and mesencephalic afferent systems. Cell body and different portions of dendrites of MSNs, are targeted by various inputs. Mainly the soma and most proximal dendrites receive recurrent collaterals from other MSN (Groves, 1983), while cortical and DAergic afferents synapse onto spines located more distally on the dendrite. On distal dendrites a significant subpopulation of spines shows a particular synaptic architecture, called “striatal microcircuit” or “synaptic triad” (Freund et al., 1984), that involves both DAergic and glutamatergic axons (Figure 2). Similar innervation architecture is also observed in pyramidal neurons in the cortex (Sesack and Pickel, 1992), hippocampus (Totterdell and Smith, 1989) and magnocellualar neurons of basolateral amygdala (Johnson et al., 1994). In this configuration, DAergic terminals make a symmetric synapse with the neck whereas cortical terminals form an asymmetric contact in the spine head (Bouyer et al., 1984; Freund et al., 1984; Smith et al., 1994). In other words, DArgic and prefrontal cortical terminals in the MSNs dually synapse on a common dendritic spine (Sesack and Pickel, 1992; Moss and Bolam, 2008). The significance of this heterosynaptic formation is not very clear but it seems to suggest that DA (Pascoli et al., 2011) is able to modulate the influence of cortical glutamatergic axons (see Spiga et al., 2014 for discussion on this point). This aspect is particularly important because, despite their distinct targets, all addictive drugs commonly abused by humans evoke variations on DA concentrations within the Nacc (Di Chiara and Imperato, 1988) and it may have a role in spine density, morphology and synaptic strength. Because of this particular synaptic configuration, even modest changes in the number of dendritic spines, can have major effects on the entire neuronal pathway. Accordingly, conditions of lowered DA tone such as morphine withdrawal has been associated with spine loss (Spiga et al., 2005). Similarly, cannabis-dependent subjects undergo spine pruning in the shell of the Nacc (Spiga et al., 2010) with a reduced MSN intrinsic excitability (Spiga et al., 2010) and alcohol-dependent rats show a DA-dependent selective loss of long thin spines associated with a lack of long term depression (Spiga et al., 2014).

## Abnormal spine plasticity and addiction

The number and shape of dendritic spines, during pathological events, are extremely variable. A broad variety of psychiatric diseases and neurological disorders are accompanied by patterns of spine disruption (Huttenlocher, 1970; Fiala et al., 2002) and changes in morphology (Irwin et al., 2000; Kaufmann and Moser, 2000). Schizophrenia, for example, is commonly associated with fewer spines and synapses in many brain areas and neuronal types (Garey et al., 1998; Glantz and Lewis, 2000; Lewis and Levitt, 2002). Further, neurodegenerative disorders such as Parkinson’s disease are characterized by a loss of dendritic spines in striatal neurons (Villalba et al., 2009). Likewise, neural events related to chronic drug intake are linked to long-lasting drug-induced whole cell plasticity (Miller et al., 2012; Diana, 2013) and abnormal spine structure and density in critical brain areas (Robinson and Kolb, 2004; Russo et al., 2010). Four functionally connected structures of the brain: medial PFC, Nacc, lateral hypothalamus and the mesencephalic VTA, represent the neuroanatomical substrate of the so-called reward pathway (Koob, 1992; Melis et al., 2005). This fundamental system of regulation of complex behavior, influences rudimentary functions like food intake (Wise, 2006), sexual behavior (Robbins and Everitt, 1996), sensory perception (Berridge and Robinson, 1998), emotions (LeDoux, 2000), intellectual evaluations and processes of memory and learning (Robbins and Everitt, 2002; Hyman et al., 2006). Drugs of abuse “illegally” occupy this circuit over-stimulating the reward mechanism, causing cumulative impacts on neurotransmission. Addictive drugs, for example, can release 2–10 times the amount of DA (Di Chiara and Imperato, 1988) that natural rewards do and they do it more quickly and more reliably. Accordingly, addiction can be considered an example of experience-dependent plasticity (Robinson and Kolb, 2004).

Drug-induced structural plasticity of dendritic spines was first described by Kunz et al. (1976) and by Riley and Halkar (1978) in hippocampal pyramidal neurons following long-term alcohol consumption and is now an emerging field of investigation (Chen et al., 2010). While chronic administration of ethanol (Zhou et al., 2007) and morphine is accompanied by a decrease in the density of dendritic spines and dendritic branching of NAcc MSNs and mPFC pyramidal neurons (Robinson and Kolb, 1999b; Robinson et al., 2002), administration (or self administration) of amphetamine (Robinson and Kolb, 1997, 1999a; Heijtz et al., 2003; Kolb et al., 2003; Li et al., 2003; Crombag et al., 2005), cocaine (Robinson and Kolb, 1999a; Robinson et al., 2001; Li et al., 2003; Norrholm et al., 2003) and nicotine (Brown and Kolb, 2001; Gonzalez et al., 2005) increases spine density and dendritic branching on NAcc MSNs and pyramidal cells in the mPFC (Kolb et al., 2003). Indeed, a direct comparison among different substances is not easy because researchers use a wide variety of doses and ways of drug administration, producing, very often, divergent results on neuron morphology, during different phases of treatment with the same substance. In particular, the withdrawal syndrome after chronic drug administration seems to be a crucial point of the addictive process that is manifested by the induction of rapid changes in dendritic spine density and morphology and is thus experimentally appealing to gain insights when the drug is not on-board, to avoid possible confounds. Accordingly, we observed radical changes on spine density in accumbal MSNs during the early phases of abstinence of various drugs of abuse (Spiga et al., 2005, 2010). In fact, spontaneous and naloxone-induced morphine withdrawal, after 14 days of escalating chronic morphine administration, selectively alters spine density in the MSN second order dendrites of the NAcc shell (Spiga et al., 2005; Diana et al., 2006). Similar results we found when rats were subjected to a chronic treatment with two different cannabinoid agonists (Delta(9)-tetrahydrocannabinol and CP 55 940) and withdrawn spontaneously and pharmacologically with the CB1 antagonist SR141716A. Confocal analysis of Golgi-Cox-stained MSNs of the NAcc revealed a decrease in spine density in the shell, but not in the core only during withdrawal (both spontaneous and pharmacologically-precipitated) (Spiga et al., 2010). In contrast, no changes in the number of spines were observed during chronic morphine, cannabis and ethanol treatment, thereby suggesting that as long as the drug is “on-board” it supports spine persistence and function, whereas abrupt withdrawal discloses spine pruning and synaptic dysfunction. Interestingly, 3 weeks of daily cocaine administration did not seem to alter spine density in the core subregion of the Nacc (Shen et al., 2009) whereas other studies showed an increased spine density in the shell (Ren et al., 2010) 1–2 days after interruption of consecutive cocaine injections in mice. Further, increases were seen in the whole Nacc (Lee et al., 2006) and the core (Kim et al., 2009). However, there are no clear indications how and whether (and if) these additional spines participate in the network activity (but see Heck et al., 2014). These experiments cast doubt and urge caution on the notion that chronic cocaine or morphine treatments are unequivocally accompanied by an increase or a decrease of dendritic spines density in the NAcc, but suggest that the withdrawal itself might be the time-window in which to observe unequivocally the reported functional and morphological changes. Indeed, chronic treatment (*per se*) without exact dosing, regimen, degree of tolerance etc., cannot offer clear-cut results. On the other hand, it should be considered that withdrawal, after (not during) chronic drug intake, is one of the most powerful factor (negative reinforcement) driving dependence (Koob and Volkow, 2010). Accordingly, it is during this phase, to expect major changes at the neural level which in turn, will elicit behavioral changes and is considered the “driving force” in the transition from chronic drug intake to “addiction” (George et al., 2014). On the contrary, repeated exposure of drugs of abuse (drug on-board) likely alters the brain, but adaptive mechanisms intervened over the course of treatment may hide objective observations, potentially misleading judgement and spoiling conclusions (Kosten and George, 2002) because, mainly due to the wide variety of drugs, diverse treatment regimens, ample dosing, different pharmacokinetic properties, and various degrees of adaptive mechanisms such as tolerance, sensitization and others.

One possible explanation for these conflicting results, is provided by the particular nature of dendritic spines, relationships with afferents and their dynamic nature in changing size, shape and function (Kasai et al., 2010). For example, in the striatum the loss of DA terminals, in animal models of Parkinson disease (Schintu et al., 2009) and/or aging (Darbin, 2012), on the spine neck removes a modulatory influence that determines if cortically derived signals invade the dendritic shaft (Garcia et al., 2010). Conversely, a decrease in activity results in elongation of spines and a collapse of their heads (Segal, 2010) or a loss altogether (Nägerl et al., 2004). Remodeling in size and morphology of dendritic spines seems to be important at least as much as their changes in density on behavioral plasticity (Grutzendler et al., 2002; Trachtenberg et al., 2002). In drug addiction (Dumitriu et al., 2012) and schizophrenia (Faludi and Mirnics, 2011), in some brain areas, spines are approximately 30% smaller than controls (Roberts et al., 1996). Two spine types seem to be particularly involved in excitatory synaptic activity: long thin and mushroom. Mushroom are large and more stable spines that can persist for months (Bourne and Harris, 2007), whereas long thin seem to be “designed” for rapid responses to changes imposed by salient stimuli (Matsuzaki et al., 2004). Although long thin spines can change their volume even independently from synaptic activity, reflecting a native instability of these structures (Yasumatsu et al., 2008), the stimulation of a single spine cause a nearly immediate expansion of the spine head volume by 3–4-fold (Matsuzaki et al., 2004). During the course of cocaine treatment, spines shift from small to large (Shen et al., 2009) as a consequence of changes in synaptic strength (Bourne and Harris, 2007). On the contrary, thin spines shift toward smaller size in response to cocaine withdrawal with the addition of new thin spines (Dumitriu et al., 2012), perhaps immature, and silent synapses (Huang et al., 2009), that contain NMDA but few or no AMPA receptors (Russo et al., 2010). These newly formed spines appear to be highly “plastic”, being able to retract or consolidate into larger spines (Shen et al., 2009). Therefore, the stabilization of heads enlargement of potentiated spines is associated with recruitment of additional AMPA-type glutamate receptors (Nusser et al., 1998; Kharazia and Weinberg, 1999) and an increase of protein synthesis as well as actin remodeling (Matsuzaki et al., 2004; Okamoto et al., 2004; Bramham, 2008; Honkura et al., 2008). In line with an active remodeling theory, by the introduction of a new staining method combining Golgi-Cox impregnation with immunofluorescence (Spiga et al., 2011), we recently found that the reduction in spine density in ethanol abstinent rats could be attributed almost entirely to long thin spines (while “mushroom” remains relatively unaffected) (Spiga et al., 2014). At the same time, PSD-95 and tyrosine hydroxilase (but not DA transporters) immunoreactivity were similarly reduced in association with ethanol withdrawal. These results show a close relationship between morphology and function of spines and reiterate on the trophic role of DA on spines in addictive states (Melis et al., 2005; Diana, 2011) and further support the “hypodopaminergic state” as a key element in animal models of addiction. On the other hand, long thin spines, in MSNs, could be strategically used as elements highly modifiable to support important modulatory roles in synaptic transmission (Jones, 2011).

It seems clear that even a single neuron respond differently as a result of exposure to different drugs and different modality of intake of the same drug in a sort of learned addictive behavior or “memory of addiction” (Mello, 1972; Kalant, 1973; Boening, 2001; Nestler, 2013; Dong and Nestler, 2014). This kind of “memory” may be similar to the long-term learning model supported by excitatory synapses located on dendritic spines (Kasai et al., 2010) of neurons in the dopaminoceptive areas such as PFC and hippocampus. This raises the possibility that long lasting changes in synapse formation and synaptic organization induced by drugs of abuse, may interact and hinder those produced by experience in the reward pathway. These drug-paired memories and the drug withdrawal-associated aversive feeling have been suggested to contribute to the high rate of relapse among addicts (Nestler, 2001; Hyman et al., 2006; Robbins et al., 2008). This wrong (aberrant) learning mechanism should be strongly related to synapse formation, changes in efficacy of synaptic transmission and morphology, modulated by DA tone in different cell types and brain regions. The resulting changes in neuronal connectivity are likely to contribute to hamper cognitive functions such as decision making and emotional rigidity typical of addicts.

## Conflict of interest statement

The authors declare that the research was conducted in the absence of any commercial or financial relationships that could be construed as a potential conflict of interest.
